# Impact of Plasminogen Activator Inhibitor-1 Serum Levels and the -675 4G/5G Variant in the SERPINE1 Gene on Systemic Sclerosis in a Mexican Population

**DOI:** 10.3390/life14091056

**Published:** 2024-08-23

**Authors:** José Alvaro Lomelí-Nieto, José Francisco Muñoz-Valle, José Eduardo Navarro-Zarza, Christian Johana Baños-Hernández, Jesús Alberto Gutierrez-Brito, Valeria Renteria-Cabrera, Eduardo Arturo Horta-Chávez, José Javier Morales-Núñez, Samuel García-Arellano, Isela Parra-Rojas, Jorge Hernández-Bello

**Affiliations:** 1 Instituto de Investigación en Ciencias Biomédicas, Centro Universitario de Ciencias de la Salud, Universidad de Guadalajara, Guadalajara 44340, Mexicodrjosefranciscomv@cucs.udg.mx (J.F.M.-V.); javier.morales@academicos.udg.mx (J.J.M.-N.);; 2 Departamento de Medicina Interna-Servicio de Reumatología, Hospital General de Chilpancingo “Dr. Raymundo Abarca Alarcón”, Chilpancingo de los Bravo 39020, Mexico; 3 Facultad de Ciencias Químico-Biológicas, Universidad Autónoma de Guerrero, Chilpancingo 39020, Mexico; iprojas@yahoo.com

**Keywords:** PAI-1 in SSc, *SERPINE1* 4G/5G polymorphism, soluble PAI-1 in autoimmune diseases, TGF-β and PAI-1 in SSc, PAI-1 and fibrotic diseases

## Abstract

Systemic sclerosis (SSc) is characterized by a complex interplay of vascular damage, inflammation, and fibrosis, affecting the skin and internal organs. Plasminogen activator inhibitor-1 (PAI-1), a protein encoded by the *SERPINE1* gene, is a potential biomarker of SSc because it is primarily involved in fibrinolysis and is associated with the severity of some autoimmune diseases. This study aimed to determine the association between *SERPINE1* variant -675 4G/5G and soluble PAI-1 (sPAI-1) levels with the clinical characteristics and risk of SSc in a Mexican population. This cross-sectional study included 56 SSc patients and 114 control subjects (CSs). The variant was genotyped via the PCR–RFLP method and the levels of sPAI-1 were determined using enzyme-linked immunosorbent assays (ELISAs). The -675 4G/5G variant was not associated with SSc risk or sPAI-I levels. However, higher sPAI-1 levels were observed in SSc patients than in CSs (*p* = 0.045); these levels were significantly correlated with age, platelets, glucose, and serum levels of transforming growth factor (TGF)-β1, 2, and 3. The *SERPINE1* -675 4G/5G variant did not show any association with SSc risk or sPAI-I levels. However, our study shows a possible alteration of sPAI-1 in this disease, which could be associated with the fibrotic and thrombotic processes in SSc.

## 1. Introduction

SSc is a chronic autoimmune disease characterized by microangiopathy, immune dysfunction, and fibrotic changes involving the skin and internal organs [1]. Clinically, SSc presents with a wide array of symptoms impacting the dermatological, respiratory, cardiovascular, musculoskeletal, renal, and neurological systems [2]. Despite its relatively low prevalence of 3 to 24 cases per 100,000 inhabitants globally [3], it is recognized for its significant morbidity and mortality, mainly due to pulmonary complications that reduce survival rates [4].

The etiology of SSc is not yet fully understood; however, it is classified as a multifactorial disease [5]. Vascular injury and endothelial dysfunction are crucial to its development, triggering uncontrolled inflammatory responses and contributing to tissue fibrosis and organ failure [6].

The fibrinolytic system is central to maintaining vascular health and has been implicated in SSc through observations of dysregulated functions, leading to hypercoagulation and persistent fibrin deposits [7]. Plasminogen activator inhibitor-1 (PAI-1), a member of the serpin family (serine protease inhibitors), is central to this system. PAI-1 plays a crucial role in regulating the plasminogen/plasmin system by inhibiting tissue-type (tPA) and urokinase-type (uPA) plasminogen activators, thereby contributing to fibrin persistence [8]. Therefore, PAI-1, in conjunction with other coagulation factors such as von Willebrand or matrix metalloproteinase 12, is considered a causative factor in some progressive fibrotic disorders, particularly in the context of pulmonary fibrosis, where transforming growth factor (TGF)-β appears to be increased due to excessive extracellular matrix deposition and reduced matrix breakdown, which are important in the pathophysiology of systemic sclerosis [9].

Elevated PAI-1 concentrations have been linked to the development of tissue fibrosis in multiple organs, including the skin, lungs, kidneys, and liver [10]. PAI-1 is produced by various types of cells, such as endothelial cells, adipocytes, macrophages, cardiomyocytes, fibroblasts, megakaryocytes, hepatocytes, and platelets [11], all of which are altered in SSc. Various factors such as TGF-β, TNF-α, and platelet-derived growth factor (PDGF) stimulate the production of PAI-1 [12,13,14]; therefore, it could be overexpressed in diseases involving inflammatory, fibrotic, and vascular complications such as SSc. However, the specific role of PAI-1 in autoimmune diseases is currently under debate due to discordant results being reported [14].

Additionally, the insertion/deletion -675 4G/5G variant (rs1799889), located in the promoter region of the *SERPINE1* gene (which encodes PAI-1), has been identified as a potential biomarker for susceptibility to inflammatory, cardiovascular, autoimmune, and fibrotic diseases [15,16,17,18]. This variant has been associated with PAI-1 activity, especially under stress conditions [19]; in particular, increased PAI-1 activity is related to the 5G allele, which binds the upstream regulatory factor (USF-1) with lower efficiency than the 4G allele [20]. Therefore, considering the crucial role of PAI-1 in fibrosis and its increased expression in the presence of the 4G allele, there might be a potential for more severe disease progression in SSc patients carrying this allele.

In the context of the Mexican population, the prevalence and pathophysiological understanding of SSc is notably limited, underscoring a significant gap in research. Addressing this, our study explores the association between the -675 4G/5G variant in the *SERPINE1* gene and sPAI-1 levels in SSc patients from Mexico.

## 2. Materials and Methods

### 2.1. Subjects

This study included 56 SSc patients and 114 control subjects (CSs). SSc patients were diagnosed by a rheumatologist according to the 2013 ACR/EULAR classification criteria [21], and recruited from the Department of Internal Medicine/Rheumatology of the General Hospital of Chilpancingo “Dr. Raymundo Abarca Alarcón”, in the state of Guerrero, Mexico.

The CSs group comprised healthy individuals (identified via self-reporting) recruited from the general population. SSc patients and CSs were unrelated individuals from the same population. To prevent population heterogeneity, only Mestizo subjects from Southern Mexico (specifically from the state of Guerrero) were included. These individuals were traceable for at least three generations and shared a similar ancestry with the SSc group.

The exclusion criteria included individuals with any autoimmune diseases, coagulation disorders, or history of cardiovascular diseases, and anyone undergoing treatments known to affect PAI-1 levels (e.g., taking anticoagulants, oral contraceptives, or antihypertensive medications). Pregnant or breastfeeding women, individuals with active infections, and subjects with renal diseases were also excluded. Informed written consent was obtained from all subjects before their enrollment in the study, in accordance with the Declaration of Helsinki ethical guidelines. This investigation was approved by the Ethics Committee of the Hospital General de Chilpancingo Dr. Raymundo Abarca Alarcón, Chilpancingo de los Bravo, State of Guerrero, Mexico.

### 2.2. DNA Extraction and Genotyping

Genomic DNA (gDNA) was extracted from leukocytes obtained from whole peripheral blood samples, using the salting-out method [22]. The -675 4G/5G variant was determined using the polymerase chain reaction–restriction fragment length polymorphism (PCR-RFLP) method. The PCR process was performed in a total volume of 25 µL. This mixture included 1 µg of DNA, 1.25 U/µL of Taq DNA polymerase with its corresponding buffer at 1X concentration, 1.5 mM of MgCl_2_, and 0.1 mM of each deoxynucleotide triphosphate (dNTP), supplied by Invitrogen™ Life Technologies, Carlsbad, CA, USA. Additionally, 0.06 µM of each primer was used, with the forward primer sequence being 5′-CACAGAGAGAGTCTGGCCACGT-3′ and the reverse primer sequence being 5′-CCAACAGAGGACTCTTGGTCT-3′. These amplification conditions have been described previously by our research group [12].

The amplified fragments were subjected to enzymatic digestion using 3 U of Bsl I restriction enzyme from New England Biolabs (Ipswich, MA, USA), for 2 h and 30 min at 55 °C. The resulting fragments were then analyzed via 6% polyacrylamide gel electrophoresis and stained with silver nitrate for visualization. In this process, digestion fragments of 77 and 22 bp indicated the wild-type (5G/5G) genotype, while fragments of 98, 77, and 22 bp signified the heterozygous (4G/5G) genotype. The presence of a single 98 bp fragment denoted the homozygous (4G/4G) genotype. To confirm the reproducibility and reliability of the results, the genotyping of polymorphisms was carried out in duplicate in 25% of the samples.

### 2.3. Levels of Soluble PAI-1 and Soluble TGF-β (sTGF-β) Isoforms

PAI-1 antigen levels were measured in serum samples from SSc patients and CSs using the Human PAI-1 ELISA Kit (Invitrogen, Carlsbad, CA, USA; Cat. KHC3071), according to the manufacturer’s instructions. The assay’s analytical sensitivity was <30 pg/mL. Serum levels were calculated using the corresponding recombinant human PAI-1 from a standard curve.

Soluble TGF-β (sTGF-β) isoform levels were sourced from our database as originally quantified in a prior study by our research group [23]. These levels were then quantified in serum using the “Bio-Plex Pro™ TGF-β Assays” multiplex test (Bio-Rad Laboratories, Hercules, CA, USA), which employs magnetic beads, using the methodology detailed in the referenced study.

### 2.4. Autoantibodies

The presence of antinuclear autoantibodies (ANAs) was determined in serum using an indirect immunofluorescence assay (IIFA) in human epithelial cells (HEp-2) according to the instructions provided by the manufacturer (BioSystems, Barcelona, Spain). Visual pattern recognition was performed on the HEp-2 cell slides, and patterns were established in accordance with the International Consensus on Antinuclear Antibody Standards (ICAP) recommendations. The following autoantibodies were assessed in all SSc patients using a second-generation ELISA: anti-topoisomerase (anti-Scl70, BioSystems, Cat. No. COD44863), anti-centromere (CENPB, BioSystems, Cat. No. COD44865), anti-fibrillarin (AFA, CUSABIO, Wuhan, Hubei, China; Cat. No. CSB-E09697h), and anti-RNA polymerase III (anti-RNA PolIII, CUSABIO, Wuhan, Hubei, China; Cat. No. CSB-EQ027833HU).

### 2.5. Statistical Analyses

Statistical analyses were performed using GraphPad Prism v8.0. The Shapiro–Wilk normality test was applied to verify the normal distribution of the data. For descriptive analyses, categorical variables were expressed as frequencies; continuous variables with nonparametric distribution were expressed as medians and percentiles (5th–95th); and parametric variables were expressed as the mean ± standard deviation (SD). All data compared between groups showed a nonparametric distribution; therefore, the Mann–Whitney U test was used to evaluate the differences between quantitative determinations of the two groups, and Fisher’s exact test was used to compare the qualitative variables. For allelic distribution analysis, the χ^2^ test was used to compare groups. Additionally, the Hardy–Weinberg equilibrium was evaluated by conducting the χ^2^ test in the control group. A *p*-value < 0.05 was considered statistically significant.

## 3. Results

### 3.1. Clinical and Demographic Characteristics

The demographic and clinical data of the SSc patients and CSs are shown in Table 1. The main results of the demographic data analysis showed no significant differences between genders or ages, indicating that these parameters did not interfere with the subsequent analysis. Regarding the SSc subtype, 48 patients were observed with limited cutaneous systemic sclerosis (lcSSc) and 8 with diffuse cutaneous systemic sclerosis (dcSSc). The mean age at the onset of SSc was 41 ± 16 years, and the median evolution time of the disease was 7.9 years. Regarding treatment, 50% of the patients were treated with methotrexate, 36% with prednisone, and 13% with chloroquine, while 38% were not undergoing treatment for their condition, as they were newly diagnosed patients.

### 3.2. Variant Distribution in SSc Patients and CSs

The Hardy–Weinberg equilibrium for the -675 4G/5G variant in the CS group showed no deviation (*p* > 0.05); therefore, this genetic variant was considered to be in equilibrium in the study population. Table 2 shows the analysis results for the genetic variant, considering the codominant, dominant, recessive, and allele inheritance models. Across these models, the frequencies of alleles and genotypes were very similar between patients with SSc and CSs; therefore, no significant association was found (*p* > 0.05).

### 3.3. Soluble Levels of PAI-1 in SSc Patients and CS

**Figure 1:**
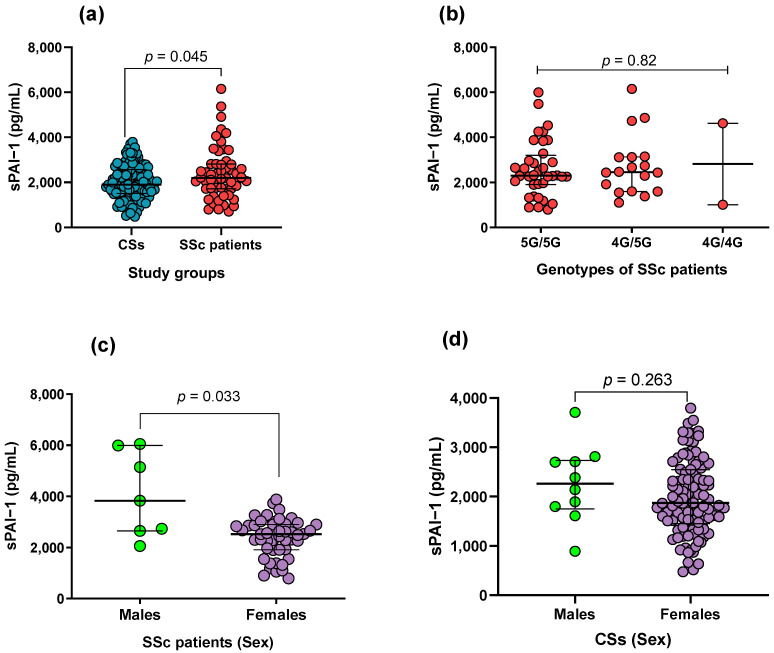
Comparison between sPAI-1 levels and different groups. (**a**) A significant difference was found between the SSc and CSs groups. (**b**) No significant difference was found between genotypes in SSc patients. (**c**) A significant difference was found between male and female patients. (**d**) No significant differences were observed in relation to gender in the control group. Dots represent individual patients, central line indicates median, and box shows interquartile range (IQR). Graphic created with GraphPad Prism v8.0.

The PAI-1 levels were higher in SSc patients than in the CSs (*p* = 0.045; Figure 1a). However, no significant difference in PAI-1 levels was observed when comparing patients by genotype (Figure 1b). On the other hand, a significant difference in PAI-1 levels between male and female SSc patients was found (*p* = 0.033; Figure 1c), but this difference was not found in the CSs (Figure 1d).

### 3.4. SERPINE1 Genetic Variant and Clinical Parameters of SSc Patients

The clinical characteristics and laboratory values were evaluated across different genotypes of the PAI-1 -675 4G/5G variant; however, no significant association was observed in SSc patients.

### 3.5. Association of sPAI-1 Levels with Clinical and Laboratory Characteristics

**Figure 2:**
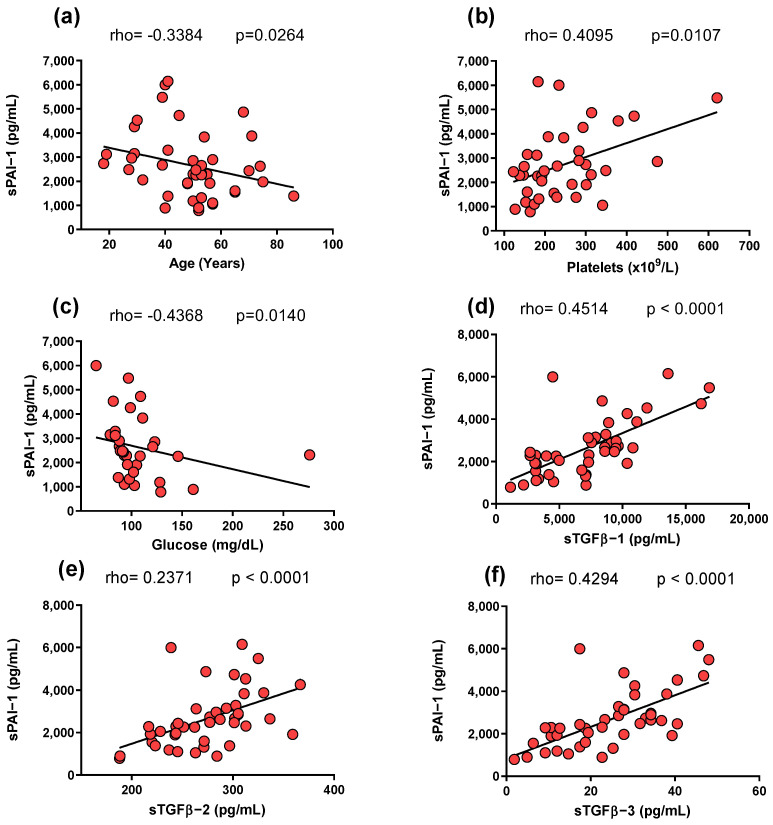
Comparison of sPAI-1 with clinical and paraclinical characteristics in SSc patients. (**a**) A negative correlation was found between sPAI-1 and aging. (**b**) A positive correlation was found between sPAI-1 and platelets. (**c**) A negative correlation was found between sPAI-1 and glucose. (**d**–**f**) A positive correlation was found between sPAI-1 and sTGF-β1, 2, and 3, individually. Correlation analyses were conducted using Spearman’s correlation coefficient. The red dots represent the individual data points for the two variables being analyzed, while the black line represents the trend line according to Spearman’s correlation coefficien. Graphic created with GraphPad Prism v8.0.

The sPAI-1 levels were negatively correlated with age (rho = −0.3384; *p* = 0.0264; Figure 2a) and circulating glucose level (rho = −0.4368; *p* = 0.0140; Figure 2c). Conversely, a positive correlation was found with platelets (rho = 0.4095; *p* = 0.0107; Figure 2b). Regarding cytokines, a positive correlation was found between sPAI-1 levels and all three isoforms of TGF-β (*p* < 0.05; Figure 2d–f).

**Figure 3:**
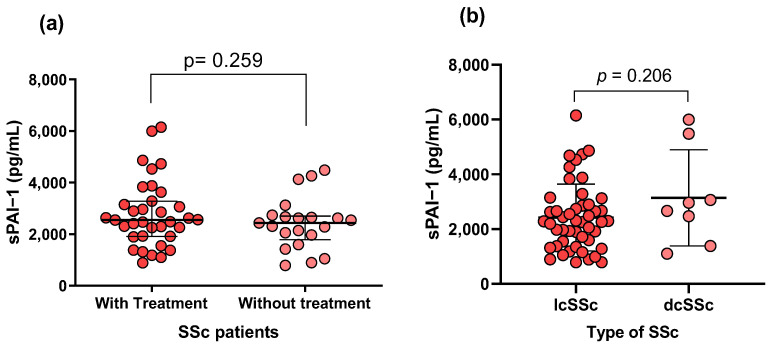
Comparison of PAI-1 levels in SSc patients based on treatment status and disease subtypes. (**a**) PAI-1 levels in SSc patients comparing those receiving treatment versus those without treatment; (**b**) PAI-1 levels across different subtypes of SSc, including limited (lcSSc) and diffuse (dcSSc) forms. No significant differences were found. Dots represent individual patients, central line indicates median, and box shows interquartile range (IQR). Graphic created with GraphPad Prism v8.0.

As for the impact of treatment, no significant differences were detected between patients receiving conventional therapy and those not receiving treatment (*p* = 0.259; Figure 3a), or among the different SSc subtypes (*p* = 0.206; Figure 3b). The comparative analysis between SSc subtypes with the clinical characteristics, PAI-1 levels, and genotypes also did not show significant differences.

## 4. Discussion

Some reports support the involvement of PAI-1 in autoimmunity, such as findings from synovial biopsies from SSc patients, which reported the presence of inflammatory cells and fibrin deposits [24]. Additionally, fibrinolytic enzymes and elevated PAI-1 levels have also been observed in the synovial fluid of patients with rheumatoid arthritis (RA) [25]. Therefore, intra- or extra-articular fibrin formation could occur due to elevated thrombogenic factors such as PAI-1 [26].

In the present study, increased levels of sPAI-1 were observed in SSc patients compared to CSs (Figure 1a). This differs from a previous study that reported decreased plasma levels of PAI-1 in SSc patients compared to CSs [14]. This discrepancy might be due to differences in the samples analyzed (serum or plasma) or patient populations studied, including the stage and severity of the SSc, demographic factors, or underlying genetic predispositions. Furthermore, the role of PAI-1 in the pathogenesis of SSc could be more complex than previously understood. While PAI-1 is known to be involved in fibrosis and vascular abnormalities—key features of SSc—its exact role might vary depending on the dynamic interplay of other pathophysiological factors in each individual. For example, PAI-1’s interaction with other cytokines and growth factors, which can vary among patients, might influence plasma levels [13]. Moreover, the previous study [14] involved SSc patients that were older than the individuals in the control group, potentially impacting the results. This factor is particularly relevant as our study observed a negative correlation between PAI-1 levels and age (Figure 2a). However, it is essential to note that further research is needed to confirm this observation, as limited data have evaluated soluble PAI-1 levels in SSc.

In autoimmune pathologies, studies on the role of soluble PAI-1 have produced discordant results. One meta-analysis revealed significantly higher circulating sPAI-1 levels in patients with systemic lupus erythematosus (SLE) compared to a control group, and similar elevations were observed in RA patients [27]. However, this pattern is not consistent across all studies; some studies have reported no significant differences between patients and CSs in atherosclerosis, RA, and SLE [12,28,29].

In another study, PAI-1 levels were analyzed in the skin of dcSSc and lcSSc patients using immunohistochemistry. This study extended to an in vivo murine fibrosis model and in vitro tests on human microvascular endothelial cells to investigate PAI-1’s role in SSc pathology. Key findings included elevated PAI-1 levels in the epidermis and endothelium of SSc patients. Notably, PAI-1 neutralization led to significant improvements in skin condition, vasculopathy resolution, reduced inflammation, and decreased fibrosis in the murine models [30]. These results suggest that targeting PAI-1’s antifibrinolytic function could be a crucial strategy in treating skin fibrosis in SSc, highlighting its significance in the disease’s progression.

On the other hand, the levels of von Willebrand factor, PAI-1, and matrix metalloproteinase 12 were elevated in lung endothelial cells isolated from a murine bleomycin-induced pulmonary fibrosis model. These biomarkers were also associated with higher levels of TGF-β expression, connective-tissue growth factor expression, and platelet-derived growth factor-C expression. Therefore, the increased levels of PAI-1 suggest a direct involvement in the fibrotic process, possibly by promoting excessive extracellular matrix deposition and reducing matrix breakdown, which are key features of pulmonary fibrosis [9].

In another study using an in vivo murine scleroderma model induced by bleomycin, researchers observed that a deficiency in PAI-1 led to the development of dermal sclerosis. Conversely, the presence of PAI-1 seemed to play a significant role in extracellular matrix (ECM) metabolism; however, it did not appear to prevent fibrosis. This finding suggests that while PAI-1 is involved in managing ECM components, its role may not extend to inhibiting the progression of fibrosis [31].

Sustained PAI-1 activity may contribute to excessive collagen accumulation in various tissues, leading to tissue fibrosis [32]. Additionally, PAI-1’s involvement in the TGF-β signaling pathway, through its suppression of plasma fibrinolytic activity and participation in the fibrotic processes, is also likely [33,34]. Our group has previously associated TGF-β with susceptibility to SSc, and we have reported high levels of this cytokine in the skin of SSc patients [23]. The present study observed a correlation between PAI-1 and TGF-β isoforms (Figure 2d–f); therefore, this information supports the notion that these markers promote inflammatory and profibrotic processes [35].

TGF-β is a well-known inducer of PAI-1 expression, contributing to the suppression of fibrinolysis and promoting fibrotic processes. The TGF-β1-stimulated expression of PAI-1 in vascular smooth-muscle cells (VSMCs) requires both the epidermal growth factor receptor (EGFR) pathway and the Rho/ROCK-dependent activation of SMAD2, indicating that PAI-1 expression is regulated by a complex network of signaling pathways [36]. The upregulation of PAI-1 in response to TGF-β can lead to excessive extracellular matrix deposition and reduced matrix degradation [37], which are hallmarks of SSc. This relationship suggests that PAI-1 not only serves as a downstream effector of TGF-β but may also act as an amplifier of the profibrotic environment in SSc. Alternative pathways could involve other cytokines and growth factors, such as PDGF, which also regulates PAI-1 levels [38] and may contribute to its overexpression in the context of SSc.

In a different comparison, we also observed higher levels of sPAI-1 in male patients than in females; this could be part of the risk factors associated with men with SSc, who generally have lower survival rates than women [39]. Based on our results, higher levels of PAI-1 may increase male patients’ susceptibility to developing more fibrotic effects, thereby contributing to a worse outcome. However, a study focusing on clarifying this hypothesis is needed.

Aging leads to a progressive decline in the efficacy of the fibrinolytic system, characterized by an observed increase in PAI-1 [40]. This change could be attributed to various age-related conditions such as obesity, emotional stress, insulin resistance, immune responses, and vascular sclerosis/remodeling [41]. However, our study found a negative correlation between PAI-1 levels and age among the patients with SSc (Figure 2a). This could indicate a bleeding disorder in these patients, although further research is required to study this association more thoroughly [42].

On the other hand, a positive correlation was observed between PAI-1 levels and platelet count (Figure 2b). This correlation is likely attributable to the production of PAI-1 in platelets [43,44]. Additionally, there was a negative correlation between sPAI-1 and circulating glucose (Figure 2c); this finding is controversial as it has been reported that high levels of circulating glucose increase PAI-1 synthesis in arterial wall cells in culture [45]. Therefore, the accuracy of this result could be compromised because we did not explore whether the patients were treated with antidiabetic drugs at the time of sampling. We compared PAI-1 serum levels between patients with and without conventional treatment (as reported in their clinical records) and found no significant differences (Figure 3). This indicates that these treatments do not significantly alter the observed association between PAI-1 and clinical variables.

One potential limitation of the present study is the absence of the direct measurement of PAI-1 in plasma, which could have provided data that were more comparative with the results of previous studies, as most previous studies have evaluated this biomarker in plasma. The coagulation that occurs during serum preparation can lead to protein degradation and the release of enzymes that may interfere with accurate measurements of PAI-1. However, the correlation of serum PAI-1 levels with different states and conditions has been thoroughly documented [46,47,48].

On the other hand, despite this variant being associated with PAI-I levels and other autoimmune and vascular diseases [12,15,18,49,50], our findings revealed no significant association between the -675 4G/5G variant in the *SERPINE1* gene and the risk of SSc or its clinical variables in the Mexican population. This outcome suggests that while PAI-1 plays a role in the pathogenesis of SSc, the specific genetic variation within the *SERPINE1* gene may not directly impact the disease’s clinical manifestation. This lack of association is a crucial observation as it indicates that other factors, possibly environmental factors or additional genetic elements, might significantly influence this disease’s progression and severity.

## 5. Conclusions

In conclusion, it becomes evident that while our study enhances the understanding of PAI-1’s involvement in SSc, particularly at the serum level, the role of specific genetic variations, such as the -675 4G/5G variant in the *SERPINE1* gene, might be less influential than previously hypothesized. Due to the scarcity of studies examining soluble levels and the -675 4G/5G variant in SSc, comparative analyses and deeper discussions on this subject remain challenging. Therefore, more research is needed to fill this gap.

## Figures and Tables

**Table 1:** Demographical and clinical characteristics of the SSc patients and CSs.

	SSc, n = 56	CSs, n = 114	*p*-Value	
**Demographic Data**				
Age (years) ^a^	50 (38–57)	47 (35–56)	0.242	
Male ^b^ Female ^b^	7 (12) 49 (88)	10 (9) 104 (91)	0.413	
**Type of SSc and Clinical Record**				
lcSSc ^b^	48 (86)	-		
dcSSc ^b^	8 (14)	-		
Age of disease onset (years) ^c^	41 ± 16	-		
Disease evolution (years) ^a^	7.9 (0.71–20.9)	-		
Signs and symptoms (n (%))				
Active Raynaud’s phenomenon ^b^	41 (73)	-		
Calcinosis ^b^	4 (7)			
Esophageal dysfunction ^b^	9 (16)			
Sclerodactyly ^b^	52 (93)	-		
Telangiectasia ^b^	33 (59)	-		
Digital ulcers ^b^	24 (43)	-		
Puffy fingers ^b^	39 (70)	-		
PAH ^b^	5 (9)			
PID ^b^	4 (7)			
Arthritis ^b^	49 (88)	-		
Clinical evaluation				
Mouth opening (cm) ^a^	5.4 (3.08–7.00)	-		
MRSS score ^a^	6.4 (0.00–27.7)	-		
HAQ-DI, 0–3 score ^a^	0.40 (0.0–1.3)	-		
VAS score ^a^	29.8 (0.0–78.5)			
ANAs ^b^	43 (77)	0 (0)		
ACAs ^b^	14 (25)	0 (0)		
Anti-Scl70 ^b^	4 (7)	0 (0)		
Anti-RNA pol III ^b^	1 (2)	0 (0)		
Anti-fibrillarin ^b^	7 (13)	0 (0)		
TGF-β1 ^a^	7307 (3905–9448)	24,386 (14,683–24,386)	*p* < 0.0001	
TGF-β2 ^a^	275.2 (243.7–304.6)	386.3 (359.1–437.3)	*p* < 0.0001	
TGF-β3 ^a^	27.25 (18.42–34.27)	49.86 (42.44–55.37)	*p* < 0.0001	
Treatment (n (%))				
NSAIDs ^b^	8 (14)	-		
Prednisone ^b^	20 (36)	-		
Methotrexate ^b^	28 (50)	-		
Chloroquine ^b^	7 (13)	-		
No treatment ^b^	21 (38)	-		

^a^ Data provided as the median (p5–p95). ^b^ Data provided as “n” (the number of individuals) and a percentage. ^c^ Data provided as the mean ± standard deviation. SSc: systemic sclerosis. CSs: control subjects. lcSSc: limited cutaneous systemic sclerosis. dcSSc: diffuse cutaneous systemic sclerosis. PAH: pulmonary arterial hypertension. PID: pulmonary interstitial disease. MRSS: Modified Rodnan Skin Score. HAQ-DI: Health Assessment Questionnaire Disability Index. VAS: Visual Analog Scale. ANAs: antinuclear antibodies. TGF: transforming growth factor. NSAIDs: non-steroidal anti-inflammatory.

**Table 2:** Genetic variant distribution in SSc patients and CSs.

Variant	Allele/Genotype	SSc, n = 56: n (%)	CSs, n = 114: n (%)	OR (CI 95%); *p*
-675 4G/5G				
Alleles	4G 5G	22 (20) 90 (80)	55 (24) 173 (76)	1.0 1.3 (0.7–2.2); 0.3
Assessed association model				
Codominant	4G/4G 4G/5G 5G/5G	2 (4) 18 (32) 36 (64)	7 (6) 41 (36) 66 (58)	1.0 1.5 (0.2–8.1); 0.6 0.8 (0.4–1.6); 0.5
Dominant	4G/4G 4G/5G + 5G/5G	2 (4) 54 (96)	7 (6) 107 (94)	1.0 1.9 (0.3–9.6); 0.4
Recessive	4G/4G + 4G/5G 5G/5G	20 (36) 36 (64)	48 (42) 66 (58)	1.0 1.7 (0.3–8.7); 0.4

SSc: systemic sclerosis; CSs: control subjects; n: number of individuals; OR: odds ratio; CI: confidence interval. The *p*-value was calculated using the chi-square test (χ^2^).

## Data Availability

The data that support the findings of this study are available from the corresponding author upon reasonable request.
